# Computational Study on the Co-Mediated Intramolecular Pauson–Khand Reaction of Fluorinated and Chiral *N*-Tethered 1,7-Enynes

**DOI:** 10.1021/acs.organomet.2c00227

**Published:** 2022-09-02

**Authors:** Jorge Escorihuela, Lawrence M. Wolf

**Affiliations:** † Departamento de Química Orgánica, Facultad de Farmacia, Universitat de València, Av. Vicent Andrés Estellés s/n, 46100Burjassot, València, Spain; ‡ Department of Chemistry, University of Massachusetts−Lowell, 1 University Avenue, Lowell, Massachusetts01854, United States

## Abstract

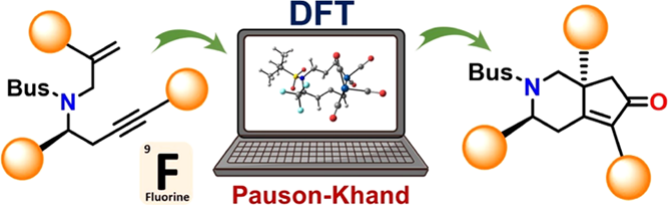

The Co_2_(CO)_8_-mediated intramolecular Pauson–Khand reaction is an elegant approach to obtain cyclopentenone derivatives containing asymmetric centers. In this work, we employed density functional theory calculations at the M11/6-311+G(d,p) level of theory to investigate the mechanism and reactivity for the Pauson–Khand reaction of fluorinated and asymmetric *N*-tethered 1,7-enynes. The rate-determining step was found to be the intramolecular alkene insertion into the carbon–cobalt bond. The stereoselectivity of the alkene insertion step was rationalized by the different transition states showing the coordination of the alkene through the *Re-* and *Si*-face. The effects of different fluorine groups and steric effects on both the alkenyl and alkynyl moieties were also theoretically investigated.

## Introduction

The Pauson–Khand reaction (PKR) is one of the elementary methodologies for the construction of cyclopentenone derivatives, which can undergo subsequent chemical transformations to access more complex structures.^1^ Since its discovery in the early 70s by Pauson and Khand,^2^ this cobalt-mediated [2 + 2 + 1] cycloaddition has become an elegant and useful transformation for the synthesis of polycyclic molecules^3^ and, in particular, for the synthesis of natural products containing the cyclopentenone motif.^4^ The PKR is generally catalyzed by cobalt, but other transition metal catalysts, such as rhodium,^5^ ruthenium,^6^ nickel,^7^ iridium,^8^ titanium,^9^ zirconium,^10^ palladium,^11^ and molybdenum,^12^ have also shown catalytic activity on this cycloaddition.

Organic fluorine compounds are of great importance in medicinal chemistry, materials science, and also as agrochemicals.^13^ Of all of them, the study of the synthesis, properties, and reactivity of monofluorinated derivatives has undergone a spectacular increase in the last two decades. As part of our ongoing studies toward the reactivity of fluorinated 1,7-enynes,^14^ we were attracted by the PKR of these starting materials,^15^ as they can afford enantioenriched nitrogenated bicycles similar to the cyclopenta[*c*]pyridin-6-one bicycle, which is present in many natural products such as tecostanine and tecomanine. On the other hand, the incorporation of fluorine atoms into biologically active molecules has proven to have beneficial effects on the stability or lipophilicity of fluorinated drugs.^16^ In terms of synthesis, the Pauson–Khand reaction of fluorinated enynes creates the bicyclic molecular complexity in just one reaction.^17^

**Scheme 1:**
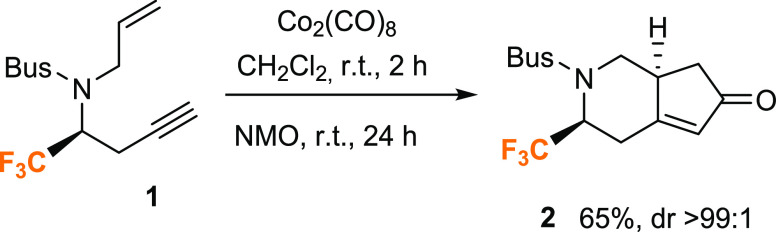
Synthesis of Cyclopenta[*c*]pyridin-6-one **2**

Recently, we reported a series of Co_2_(CO)_8_-mediated PKR for the formation of fluorinated monoterpenic alkaloid cyclopentene derivatives from chiral fluoroalkyl aldimines (Scheme 1).^18^ In this study, *tert*-butylsufonyl (Bus) *N*-protected fluorinated 1,7-enynes were used as substrates for the Pauson–Khand reaction under relatively mild reaction conditions, using dichloromethane as a solvent and *N*-morpholine *N*-oxide (NMO) as an additive. The reaction also proceeded smoothly when the methyl-substituted alkene or alkyne components were employed. Given the interest in this kind of Pauson–Khand reaction, theoretical investigations on the mechanistic details are valuable for understanding the experimental observations and for aiding further reaction design. Furthermore, when stereocenters are formed, mechanistic studies are of utmost importance to rationalize the stereoselectivity of the process.

Since the seminal theoretical work of Yamanaka and Nakamura on the intermolecular PKR,^19^ several studies on the reaction mechanism of cycloaddition have been reported.^20^ This mechanism is based on the cobalt-mediated PKR study by Magnus and Principe in 1985.^21^ This mechanism involves the initial formation of a cobalt–acetylene complex upon the reaction of acetylene and Co_2_(CO)_8_, followed by the reversible ligand exchange of CO and an alkene and then olefin insertion to yield a five-membered metallocycle. Next, carbonyl insertion takes place, followed by reductive elimination to afford a cyclopentenone complex. However, computational studies on the stereoselective intramolecular PKR of enynes are limited to only a few theoretical studies.^22^

Density functional theory (DFT) calculations have been previously employed to unravel the reaction mechanism and predict the enantioselectivity of several catalytic systems, including Pauson–Khand reactions.^23^ In this work, DFT calculations are employed to explore how fluorinated groups on the substrate influence the reactivity of the Co_2_(CO)_8_-mediated Pauson–Khand reaction of the recently reported fluorinated and asymmetric *N*-tethered 1,7-enynes. The rationalization of the regioselectivity of the process yielding a new stereocenter is supported by transition state (TS) analysis using the distortion/interaction model and noncovalent interaction (NCI) analysis. In this paper, we aim to (a) study the reaction mechanism of the newly reported asymmetric *N-*tethered 1,7-enynes having a CF_3_ group and clarify the reactivity and stereoselectivity and (b) evaluate the influence of the fluorine atom or fluorinated groups on the structure of *N-*tethered 1,7-enynes.

## Computational Details

All of the DFT calculations were carried out using the Gaussian 16 series of programs.^24^ The M11 functional^25^ with the basis set 6-311+G(d,p) for H, C, O, and N and the SDD basis set for Co^26^ in dichloromethane as a solvent (ε = 8.93) with a polarizable continuum model (PCM)^27^ was used for geometry optimizations. Harmonic frequency calculations were performed for all stationary points to confirm them as local minima or transition state structures and to derive the thermochemical corrections for Gibbs energies. Intrinsic reaction coordinate (IRC) calculations were performed to verify the expected connections of the first-order saddle points with the local minima found on the potential energy surface. The energies given in this work are M11-calculated Gibbs energies. A correction of 1.9 kcal/mol was applied to all Gibbs energies calculated to change the standard state from the gas phase (1 bar) to solution (1 M).^28^ The torsion angles were randomly varied, and the obtained structures were fully optimized. Thus, 100 minima of energies within an energy gap of 10 kcal/mol were generated. These structures were analyzed and ordered considering the relative energy, and finally, all repeated geometries were eliminated. In all cases, molecules with the lowest energy and an energy gap of 3.0 kcal/mol were selected and studied at the M11/6-311+G(d,p)&SDD level. Optimized structures were illustrated using CYLview20.3.^29^ The NCI surfaces were computed with NCIplot.^30^ Distortion/interaction analysis was performed along the reaction coordinate for comparing **1a** and **1a**′ activation energies. The data points for each geometry along the reaction coordinate were obtained by performing a relaxed scan of the breaking C–C bond from 2.99 to 1.55 Å. The total energy, distortion energy, and interaction energy for each point along the reaction path were computed at the M11/6-311+G(d,p) level of theory for the enyne substrate and at the M11/6-311+G(d,p)&SDD level for the Co catalyst. Decomposition of the interaction energy was performed using the localized molecular orbital energy decomposition analysis (LMO-EDA) within TURBOMOLE7.5.^31^

## Results and Discussion

**Figure 1:**
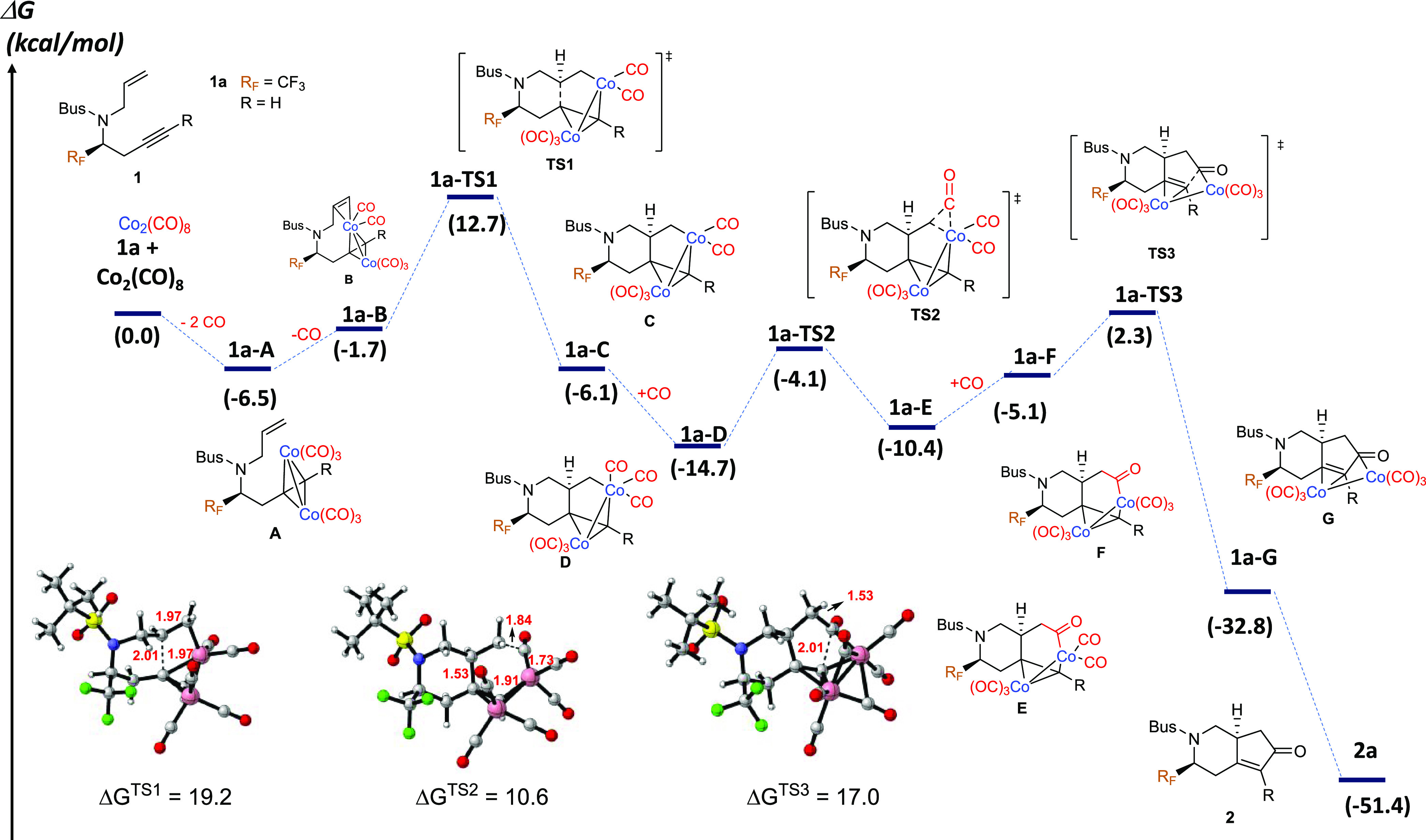
Gibbs energy profile and schematic representation of stationary points for the PKR of fluorinated *N*-tethered 1,7-enynes and optimized structures of the transition states (see Figure S1 for all optimized structures). Bond distances are given in angstroms (Å) and energies are given in kcal/mol.

We initially calculated the Gibbs energy profile for the Co_2_(CO)_8_-mediated intramolecular Pauson–Khand reaction of fluorinated enyne **1a**, and the results are summarized in Figure 1. The proposed catalytic cycle for the Pauson–Khand reaction of fluorinated 1,7-enynes is based on the traditional mechanism proposed by Magnus and Principe over 35 years ago, and the global process involves three elementary steps. The first step is the alkene insertion in which a C–C bond is formed, yielding a six-membered cycle and determining the regioselectivity of the cyclization process. In the second step, the insertion of CO on the terminal CH_2_ side of the alkene moiety takes place. The third and final step involves CO coordination followed by reductive elimination. The catalytic cycle of this reaction begins with complexation of enyne **1a** with Co_2_(CO)_8_ to form cobalt–acetylenic complex **1a-A** upon the release of two molecules of CO, as known experimentally.^32^ This transformation is moderately exergonic by 6.5 kcal/mol and entropically favored. The Co–Co distance enlarges when the number of coordinated CO ligands increases going from 2.50 Å in the initial Co_2_(CO)_8_ to 2.40 Å in **1a-A**. The intramolecular coordination of the alkene moiety to complex **1a-A** then proceeds to give **1a-B** with the release of one molecule of CO. Complex **1a-B** is thermodynamically unfavorable compared to **1a-A** but thermodynamically favorable over the starting point. Subsequent alkene insertion into the Co–C bond via transition state **1a-TS1** leads to the irreversible generation of cobaltacycle intermediate **1a-C**. The activation energy of this step is 19.2 kcal/mol with a transition state showing a C···C distance of 2.01 Å and is the rate-determining step of the PKR. The Co center generates thermodynamically favorable intermediate **1a-D** with a Gibbs energy decrease of 8.6 kcal/mol. The Co–Co distance enlarges from 2.43 Å in **1a-D** to 2.52 Å in **1a-D**, highlighting weakening Co–Co interaction with additional CO coordination. Subsequent CO insertion at the terminal C of the alkene occurs via transition state **1a-TS2** with a Gibbs energy barrier of 10.6 kcal/mol and a C···C=O distance of 1.84 Å, forming intermediate **E**. An alternative insertion of CO on the other side of the Co metal from **1a-D** yielded a TS with a higher barrier of 19.3 kcal/mol (Figure S3 in the Supporting Information.). Further, CO coordination of **1a-E** gives complex **1a-F** (5.3 kcal/mol endergonic) and is followed by rapid reductive elimination to deliver complex **1a-G** irreversibly through transition state **1a-TS3** with a C···C distance of 2.01 Å. The Gibbs energy barrier for this step from **1a-D**, which is the lowest energy point preceding **1a-TS3**, is 17.0 kcal/mol. Bicyclic product **2a** is then released from intermediate **1a-G** with the concomitant formation of Co_2_(CO)_6_ with significant exergonicity (12.2 kcal/mol).

The overall Gibbs energy of the reaction pathway is determined to be exergonic by 51.4 kcal/mol with an activation energy of 19.2 kcal/mol, which suggests that this reaction proceeds smoothly under relatively mild conditions. The step with the highest activation barrier (**1a-A** to **1a-TS1**) is 19.2 kcal/mol. The calculation results suggest that the initial alkene insertion is the rate-determining step for the overall reaction pathway, in concordance with previous studies, as observed for regular enynes but contrary to the recently reported Co_2_(CO)_8_-mediated intramolecular PKR of cyclooctene derivatives, in which the CO insertion is considered to be the rate-determining step for the overall reaction pathway.^22d^

**Figure 2:**
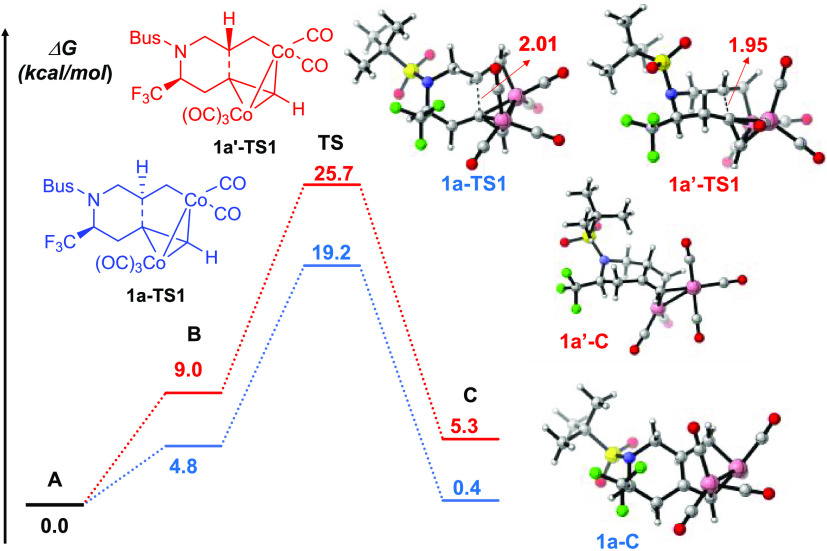
Gibbs energy profiles for the alkene insertion step of the PKR of **1**. All energies are given in kcal/mol.

As mentioned, the regioselectivity of the process is controlled along **TS1** involving the C–C bond formation and cyclization to give a six-membered ring. The calculations support the experimental formation of the product with the *S* configuration at C6 through the analysis of the competing diastereomeric transition states (Figure 2). Enyne **1a**, with the organofluorinated CF_3_ group at C4, was used to study the regioselectivity of the alkene insertion step. Coordination of the alkene through the *Re-*face in **1a-B** was found to be 4.3 kcal/mol lower than the coordination through the *Si*-face in **1a**′**-B** (Figure 2). This can be attributed to the orientation of the Bus group adopting a pseudoaxial position in the S*i*-face coordination.

A closer look at the geometry of the stationary points reveals that the Co–Co bond distance changes from 2.40 to 2.66 Å, and one Co atom is firmly bonded to the terminal C of the acetylene moiety (1.96–2.09 Å). The alkene insertion from intermediate **A** takes place via **1a-TS1** with a barrier of 19.2 kcal/mol, yielding compound **C** with the *S* configuration at C5. On the other hand, the formation of the product with the opposite configuration at C6 occurs via the later **1a**′**-TS1 (**C···C distance of 1.95 Å**)**, with a barrier of 25.7 kcal/mol, which is 6.6 kcal/mol higher than the corresponding insertion through the *Re*-face. In the new formed six-membered ring of **1a**′**-C**, the proximity between the *tert*-butylsufonyl group and CF_3_ (F···O distance of 2.63 Å and F···H distance of 2.58 Å) leads to higher relative energy. In **1a**′**-TS1**, the inserting alkenyl group is closer to a CO coordinated to cobalt. The distances between the inserting alkenyl group hydrogen and coordinated CO are 2.99 and 3.94 Å for **1a**′**-TS1** and **1a-TS1**, respectively, leading to a higher steric repulsion, and therefore, the relative free energy of **1a**′**-TS1** is higher than that of **1a-TS1**.

To compare both TSs and rationalize the different energies of the TS involved in the regioselective step (**TS1**), we applied the activation strain model (ASM), also known as the distortion/interaction model.^33^ The ASM is a helpful and complementary tool to better understand the origin of energy barriers and has been applied to a diverse range of chemical reactions, including nucleophilic substitution, elimination, cycloaddition, oxidative addition, organometallic chemistry, and other processes in organic chemistry.^34^ This model decomposes the activation barrier (Δ*E*^‡^) of a reaction into two contributions along the reaction coordinate, namely, the strain (Δ*E*_strain_^‡^) and interaction (Δ*E*_int_^‡^) energies between the fragments participating in the formation or rupture of chemical bonds. On one hand, the strain energy Δ*E*_strain_^‡^ is the energy required to deform reactants from their equilibrium geometry to reach the activated complex geometry, i.e., the transition state. Additionally, Δ*E*_strain_^‡^ can be decomposed in (i) Δ*E*_strain(enyne)_^‡^, which is the required energy to distort the enyne substrate into the transition state geometry, and (ii) Δ*E*_strain(Co)_^‡^, which corresponds to the energy to distort the Co atoms and the ligands into the transition state geometry. On the other hand, the term Δ*E*_int_^‡^ is the interaction energy between the deformed reactants in the activated complex geometry, i.e., Co_2_(CO)_5_ and enyne.

**Figure 3:**
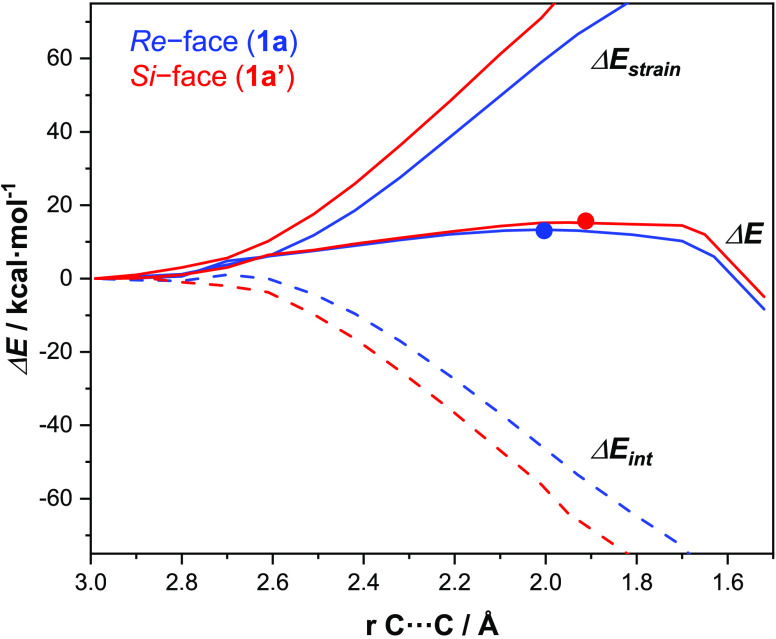
Comparison that shows the activation strain diagrams for the alkene insertion step (**TS1**) along the reaction coordinate projected onto the formed C···C bond for the PKR of enynes **1a** and **1a**′. All data were computed at the M11/6-311+G(d,p)&SDD level of theory.

The computed distortion energies of the Co catalyst (Δ*E*_strain(Co)_^‡^) in both reactions are very similar (within ±1 kcal/mol), indicating that they contribute nearly equally to each TS. Therefore, the distortion energy for the *N*-tethered 1,7-enyne is the major contribution to the distortion difference and controls the regioselectivity. As shown in Figure 3, which shows the activation strain diagrams for the two competing insertions via **1a-TS1** and **1a**′**-TS1**, the distortion of *N*-tethered 1,7-enyne **1a** corresponding to the *Re*-face attack (**1a-TS1**) is lower than that of the enyne at **1a**′**-TS1** associated with the *Si*-face attack. This energy difference is the major contribution to the difference in the activation barrier, leading to the exclusive insertion via **1a-TS1**. This large difference in the strain might suggest substantial steric-type interactions present in **1a**′**-TS1** and not present in **1a-TS1**.

**Figure 4:**
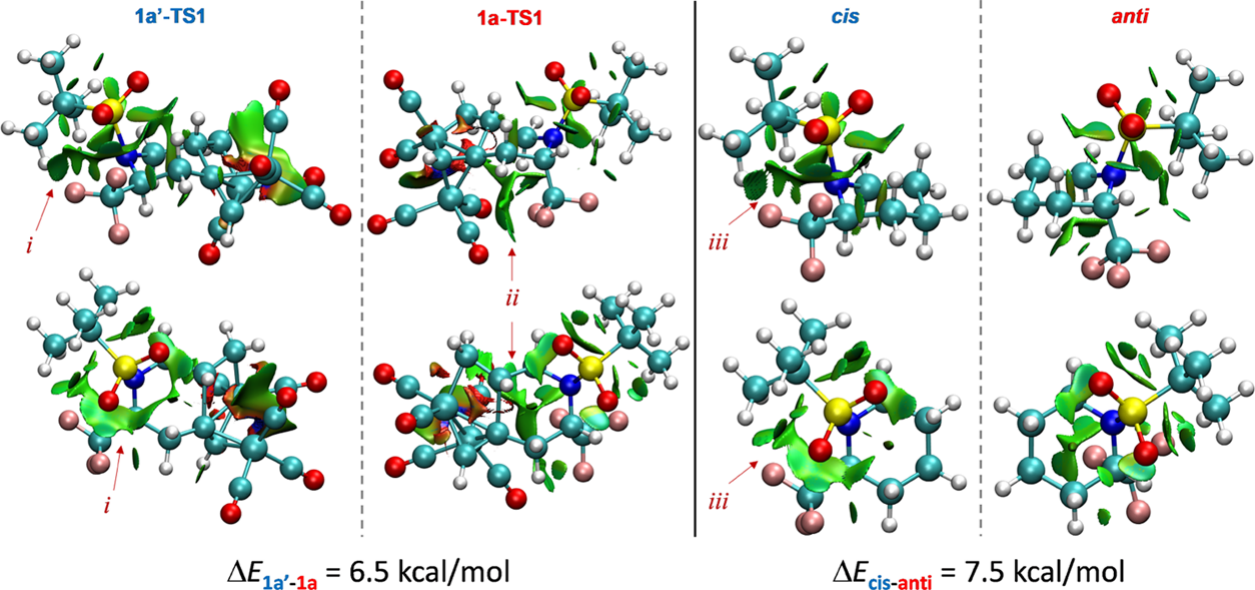
NCI surfaces of **1a**′**-TS1** and **1a-TS1** as well as a model system with both Bus and CF_3_ groups vicinal in cyclohexane ring-flip conformations. The first and second rows differ by perspective only. i–iii labels are used as descriptors in the main text.

To ascertain the role of steric-type interactions in diastereoselectivity, noncovalent interaction (NCI) analysis was performed on selectivity-determining transition states **1a-TS1** and **1a**′**-TS1** (Figure 4). Upon inspection of the major differences between the NCI surfaces, it is clear that **1a**′**-TS1** contains a significant contour between the Bus and CF_3_ groups (i), while in **1a**, there exists a similarly sized contour between the CF_3_ axial group and the Co(CO) moiety (ii), reflecting van der Waals contact. The predicted preference for **1a** over **1a**′ by 4.6 kcal/mol would suggest the CF_3_···Bus gauche-like interaction in **1a**′ to be more unfavorable than the CF_3_···Co(CO)_3_ diaxial-like interaction in **1a**, without any difference in the interaction between the π systems with the Co atoms between the two TSs. To support this inference, a model system (Figure 4, right) was probed to approximate the CF_3_···Bus gauche-like interaction by comparing two substituted chair conformations (since the actual TSs are chair-like) with the two groups being either pseudo-*cis* or pseudo-*anti*. From this difference, the CF_3_···Bus interaction (iii) can be approximated to be ca. 7.5 kcal/mol. If this interaction is extended to the TS comparison, the CF_3_···Co(CO)_3_ interaction is determined to be ca. 3.0 kcal/mol (minus a CF_3_···CH gauche interaction present in **1a-TS1**). In summary, the stereoselectivity for the overall reaction can be understood by comparing the unfavorable noncovalent interactions in the two competing transition states, with the CF_3_···Bus interaction in **1a**′**-TS1** being more than twice as costly as the CF_3_···Co(CO)_3_ interaction in **1a-TS1**.

Much of the energy differences between the two pathways are present in complex **B**. Thus, the energy decomposition analysis (EDA) of the interaction energy was performed on **1a-B** and **1**′**a-B** to reveal the energetic contributions to the complexation of Co_2_(CO)_5_ to enyne **1a**. **1a** exhibits significantly more steric repulsion than **1a**′, as reflected in the difference in the exchange-repulsion, Δ*E*_exrep_, of 18.4 kcal/mol, while also exhibiting stronger electrostatic and orbital interactions. There is also a smaller preference in the DFT correlation energy for **1a. 1a** is more strained by 3.3 kcal/mol. Much of that strain difference appears manifested structurally in the alkyne angular distortion. This strain is more than compensated for by the favorable electrostatic, orbital, and dispersion-like interactions with Co_2_(CO)_5_ to give an overall energetic preference for **1a** by 4.0 kcal/mol. The orbital preference for **1a** is significant and is probed further through a more detailed analysis of the frontier orbital interactions.

**Figure 5:**
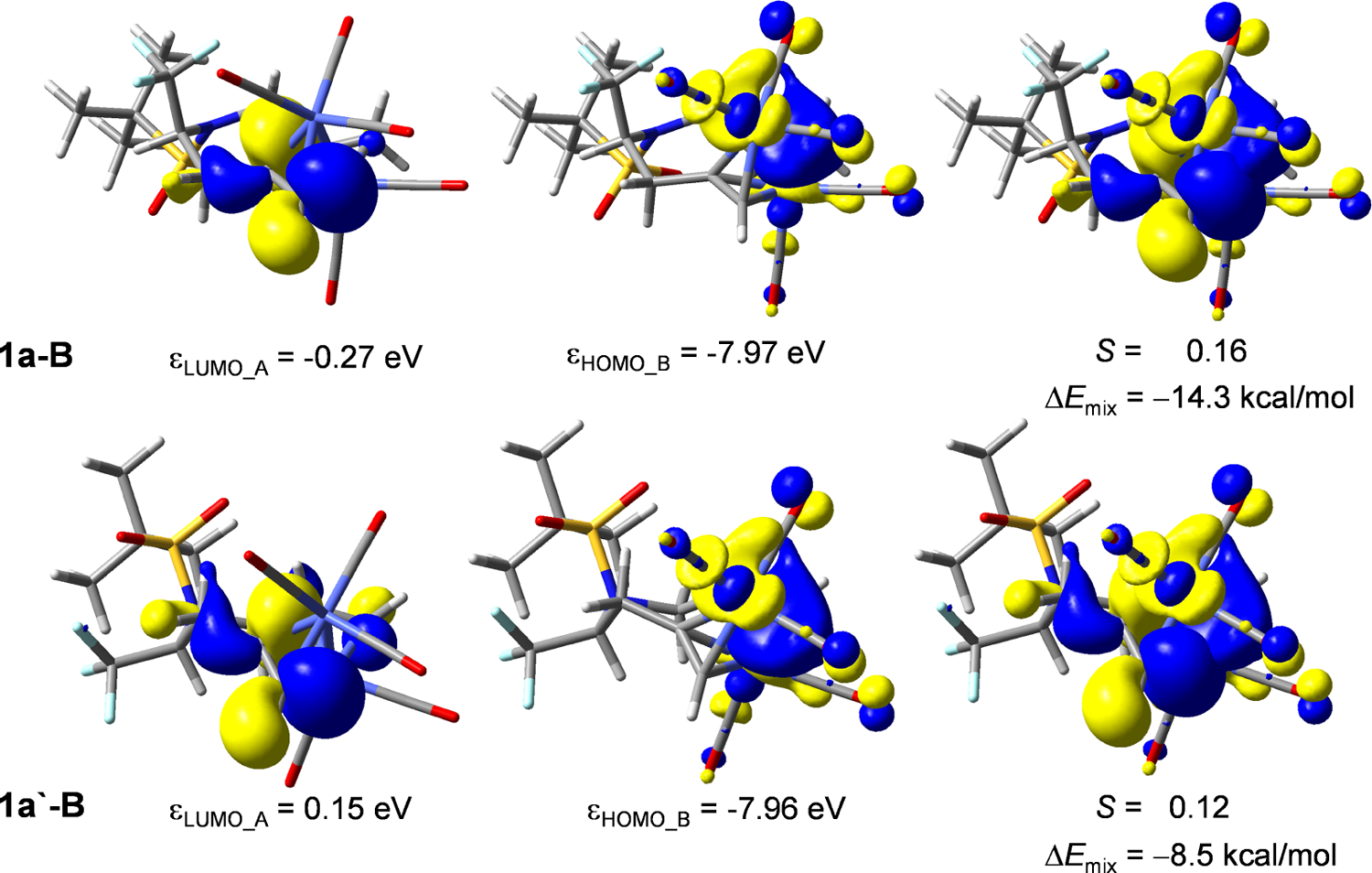
Fragment orbitals of **1A** (LUMO) and Co_2_(CO)_5_ (HOMO) in **1a-B** and **1a**′**-B** with fragment orbital energies, orbital overlap (*S*), and orbital mixing energy, Δ*E*_mix_, provided. The rightmost figure represents the overlap between the fragment orbitals.

A fragment orbital analysis was performed to probe the origin of the enhanced Δ*E*_orb_ term in **1a-B** (Table 1). The mixing interaction energies were calculated using second-order perturbation theory that includes the fragment orbital energies, orbital overlap, and Fock interaction element. The total mixing interaction (Δ*E*_mix,tot_) is calculated as a sum of mixing all occupied orbitals on the **1A** fragment mixing with the unoccupied orbitals of the Co_2_(CO)_5_ fragment (Δ*E*_mix_A → B), and vice versa (Δ*E*_mix_B → A). The computed data show that **1a-B** exhibits a greater total mixing interaction (Δ*E*_mix,tot_) by 6.1 kcal/mol compared with **1a**′**-B**. This difference is primarily manifested in the Δ*E*_mix_B → A component. The individual orbital interactions from a donation from Co_2_(CO)_5_ to **1A** were analyzed to determine the interaction with the most significant difference with the focus on the frontier orbitals. The strongest difference is present in the HOMO–LUMO interaction, with this interaction in **1a-B** (−14.3 kcal/mol) being 5.8 kcal/mol greater than that in **1a**′**-B** (−8.5 kcal/mol). These orbitals are provided for visualization (Figure 5). From this interaction, the HOMO is largely of σ type (Co–Co) on Co_2_(CO)_5_, and the LUMO is largely one of the π* orbitals of the alkyne unit of **1A**. The orbital overlap, *S*, is indeed greater in **1a-B** (*S* = 0.16) than that in **1a**′**-B** (*S* = 0.14). Perhaps more significant is the lower LUMO orbital energy in the **1A** fragment for **1a-B** (−0.27 eV) compared with that in **1a**′**-B** (0.15 eV). These orbital energy differences originate from the greater strain in the **1A** fragment by 2.6 kcal/mol (Table 1). This strain is manifested as greater angle strain in the alkyne in **1a-B** with an angle of 134° compared with that in **1a**′**-B** with an angle of 143°. The preference for **1a-B** then can be explained as having a more effective overlap between the π* orbital of the alkyne with the Co_2_(CO)_5_ HOMO resulting from the coordination of the *Re*-face of the alkene in **1a-B** than that with the *Si-*face in **1a**′**-B**. The enhanced strain in **1a-B** is more than compensated for by the greater mixing interaction between these orbitals (Table 2).

**Table 1:** Energy Decomposition Analysis of **1a** and **1a**′ Using the LMO-EDA Method^a^

	**1a-B**	**1a′-B**	**1a-B – 1a′-B**
Δ*E*_elec_	–374.3	–365.4	–8.9
Δ*E*_exrep_	652.4	634.0	18.4
Δ*E*_orb_	–263.6	–251.2	–12.5
Δ*E*_corr_	–146.4	–141.0	–5.3
Δ*E*_int_^b^	–131.9	–123.6	–8.3
Δ*E*_strain,A_	55.7	53.1	2.6
Δ*E*_strain,B_			1.7
Δ*E*_strain_^c^			4.3
Δ*E*^c^,^d^			–4.0

a (elec = electrostatic; exrep = exchange-repulsion; orb = orbital; corr = correlation). All energies are listed in units of kcal/mol.

b Δ*E*_int_ = Δ*E*_elec_ + Δ*E*_exrep_ + Δ*E*_orb_ + Δ*E*_corr_.

c Δ*E*_strain_ = Δ*E*_strain,A_ + Δ*E*_strain,B_.

d Δ*E* =Δ*E*_int_ + Δ*E*_strain_.

**Table 2:** Fragment Orbital Interaction Analysis Where Fragment A Represents **1A** and Fragment B Represents Co_2_(CO)_5_^a^

	**1a-B**	**1a**′**-B**
Δ*E*_mix_A → B^b^	–103.0	–103.6
Δ*E*_mix_B → A	–194.6	–187.9
Δ*E*_mix,tot_	–297.6	–291.5

	**1a-B**		**1a-B**′	
	LUMO	LUMO+1	LUMO	LUMO+1
HOMO	–14.3^c^	–0.2	–8.5	–1.1
HOMO-1	–17.7	–14.3	–15.0	–18.7
HOMO-2	–7.9	–0.4	–10.3	–0.4
HOMO-3	–12.2	–3.6	–15.4	–1.3
HOMO-4	–0.6	–0.1	–0.4	–0.3
HOMO-5	–13.9	–3.8	–12.8	–2.7
HOMO-6	–0.8	–4.3	–0.2	–5.1
HOMO-7	–13.8	–0.9	–10.6	–1.1

a All energies are provided in units of kcal/mol.

b Total mixing interaction from all occupied orbitals on A with all unoccupied orbitals on B. A = **1A**; B = Co_2_(CO)_5_.

c Mixing interaction between the HOMO of B with the LUMO of A.

In summary, the NCI and EDA analyses reveal that the combination of enhanced gauche steric interactions between the CF_3_···Bus groups in the **1a**′ path with the enhanced orbital mixing interactions in the *Re-*face approach of the alkene leads to a greater preference for the **1a** path. Appropriate manipulation of these interactions through careful substrate modifications could be used to elevate selectivity even further.

**Scheme 2:**
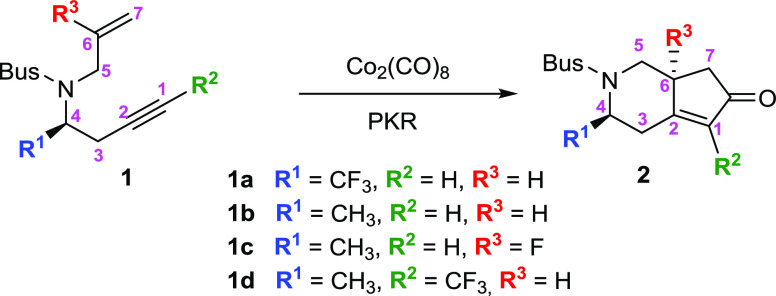
PKR of Different Fluorinated *N*-Tethered 1,7-Enynes

The influence of the fluorine atom or the fluorinated group on the enyne structure has been studied in the past decade by different authors.^35^ To further investigate the reactivity of different *N*-tethered 1,7-enynes in the Co-mediated intramolecular PKR, we calculated the Gibbs energy profiles for different 1,7-enynes bearing the fluorine atom or the fluorinated group at different positions along the enyne scaffold (Scheme 2).

**Figure 6:**
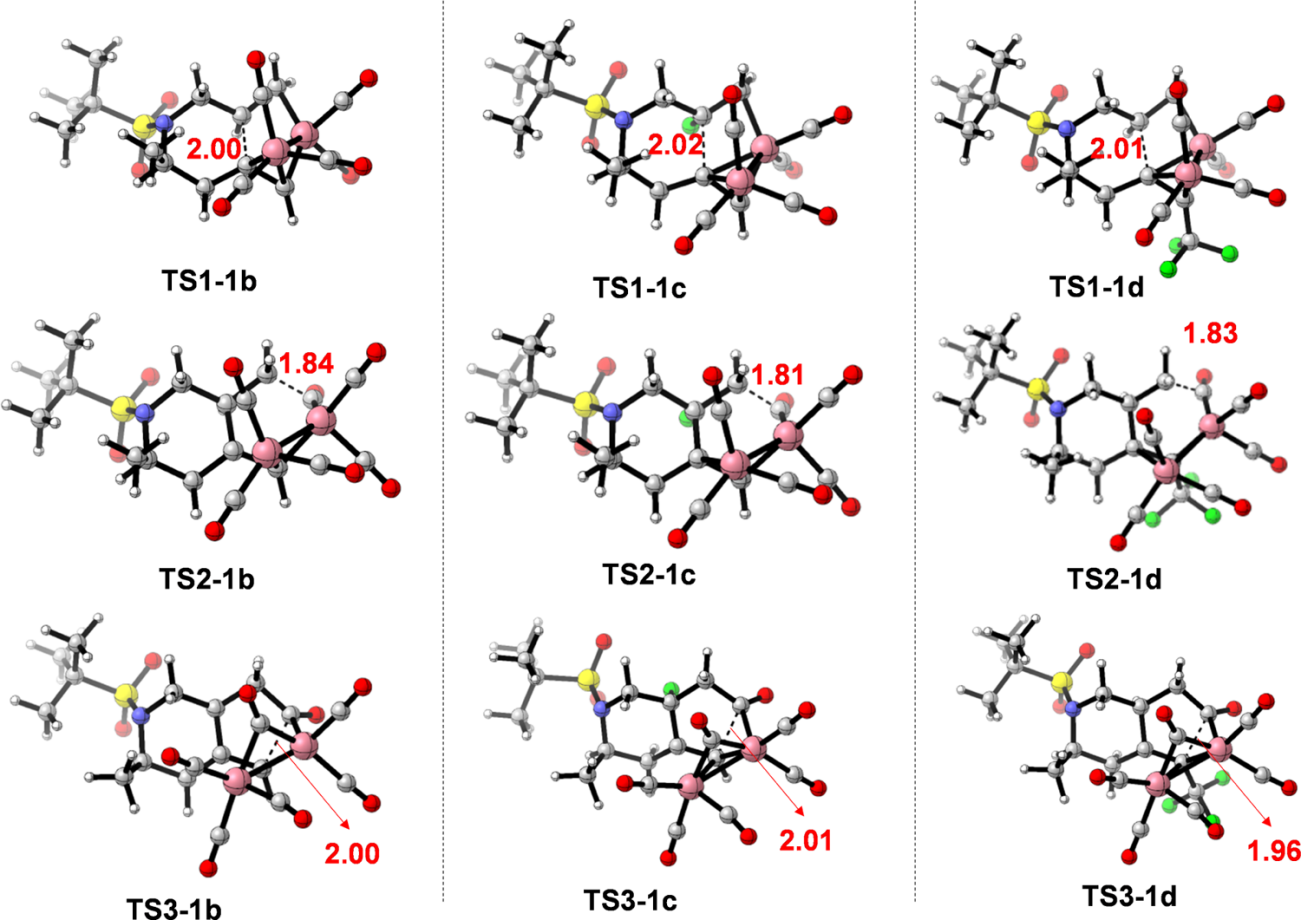
Transition states for the PKR of *N*-tethered 1,7-enynes **1b**–**d** and activation Gibbs energies in kcal/mol corresponding to the relative Gibbs energies. Bond distances are given in angstroms (Å).

The geometry information on the different transition states at the M11/6-311+G(d,p) level of theory in dichloromethane is shown in Figure 6. As inferred from Table 3, on one hand, the presence of the CF_3_ group at C4 in enyne **1a** slightly decreased the Gibbs energy of the alkene insertion step when compared to enyne **1b** bearing a CH_3_ group at the same position, favoring the rate-determining step of the process (**TS1**). On the other hand, the activation Gibbs energy of the CO insertion was found to be similar, whereas that for the reductive elimination was lower for enyne **1b**. The introduction of a fluorinated group at the asymmetric carbon of the enyne enhances the reactivity by lowering the barrier for the alkene insertion (**TS1**). On the contrary, the presence of a fluorine atom on the alkene has the opposite effect. As observed for enyne **1c**, having a F atom at alkenyl C6, a higher barrier was found for the alkene insertion when compared to enyne **1a**. Also, CO insertion and reductive elimination were found to be 1.8 and 2.5 kcal/mol higher, respectively. Both enynes **1a** and **1c** have been experimentally assayed in the Co-mediated intramolecular PKR, yielding the corresponding cyclopentenone derivatives with similar yields, 65 and 60% for enynes **1a** and **1c**, respectively.^12,17^ The regioselectivity in **TS1** involving the formation of a stereocenter was also investigated for enyne **1c** and compared to the experimental results, which showed the formation of the final bicyclic products with excellent diastereoselectivities (dr > 20:1).^15^ Theoretical results for **1c** indicated an energy difference of 4.7 kcal/mol between both TSs, which is consistent with the experimental observation of only one diastereomer (dr > 20:1). Finally, enyne **1d**, bearing a CF_3_ group at alkynyl C1, showed a different behavior in comparison with the previous derivatives. In this case, DFT calculations showed a lower activation Gibbs energy for alkene insertion (**1d-TS1**) and a slightly higher barrier for the CO insertion (**1d-TS2**) but a relatively higher penalty for the reductive elimination (22.3 kcal/mol for **1d-TS3**), associated with the electron-withdrawing effect of the CF_3_ group, thus making reductive elimination the rate-determining step of the overall process.

**Table 3:** Activation Gibbs Energies (in kcal/mol) for the TS Structures Involved in the Intramolecular PKR of Different Fluorinated *N*-Tethered 1,7-Enynes

enyne	ΔGTS1 (kcal/mol)	ΔGTS2 (kcal/mol)	ΔGTS3 (kcal/mol)
**1a**	19.2	10.6	17.0
**1b**	20.4	10.4	15.8
**1c**	21.4	12.4	20.1
**1d**	17.7	11.5	22.3

**Figure 7:**
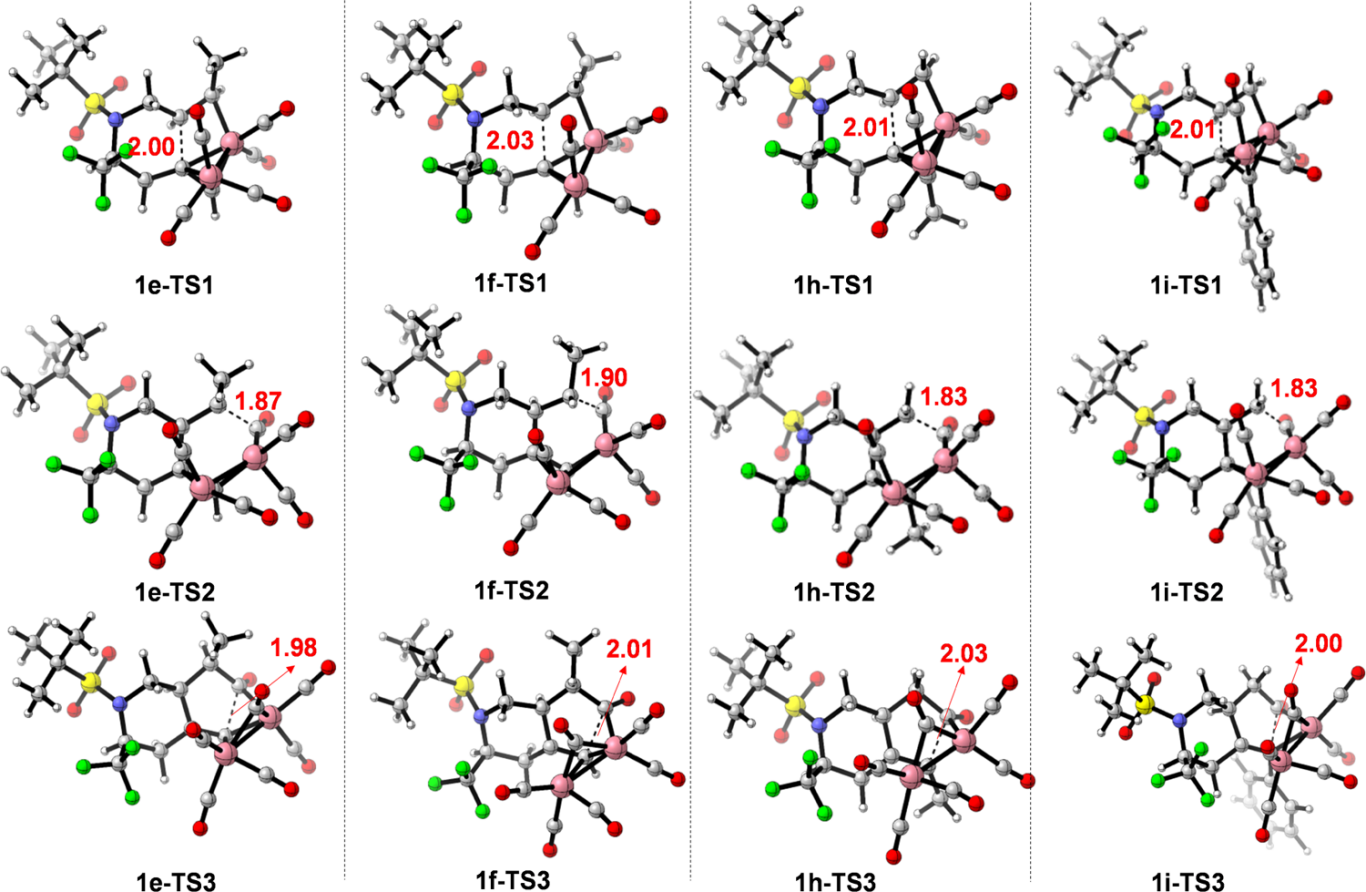
Transition states for the PKR of *N*-tethered 1,7-enynes **1f**–**1h**. Bond distances are given in angstroms (Å).

**Scheme 3:**
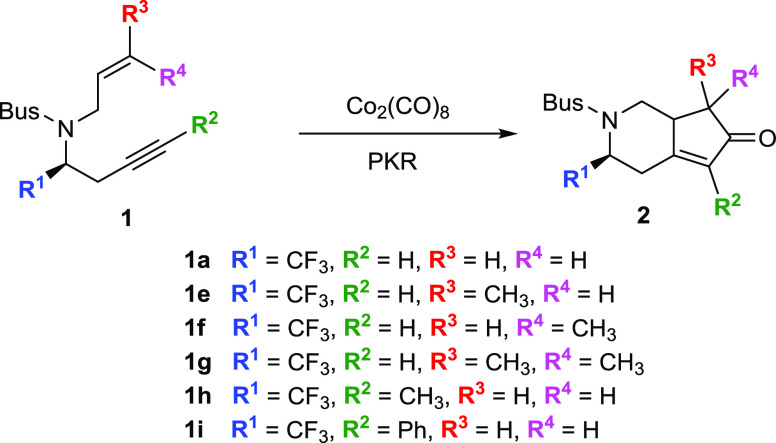
PKR of Different CF_3_ Containing *N*-Tethered 1,7-Enynes

Given the increasing interest in structures containing trifluoromethyl,^36^ we turned our attention to investigate the reactivity of different *N*-tethered 1,7-enynes containing the fluorinated CF_3_ group at C4 (Scheme 3). To this end, steric effects on both the alkenyl and alkynyl moieties were assayed by the introduction of a CF_3_ group (enynes **1e**–**1h**), and the Gibbs energy profiles for the reaction pathways were calculated at the same level of theory (M11/6-311+G(d,p) in dichloromethane as a solvent). The optimized structures on the different transition states are shown in Figure 7, and the relative Gibbs energies for each TS are listed in Table 4.

**Table 4:** Activation Gibbs Energies (in kcal/mol) for the TS Structures Involved in the Intramolecular PKR of Different Fluorinated *N*-Tethered 1,7-Enynes

enyne	Δ*G*^TS1^(kcal/mol)	Δ*G*^TS2^(kcal/mol)	Δ*G*^TS3^(kcal/mol)
**1a**	19.2	10.6	17.0
**1e**	23.4	11.3	12.4
**1f**	22.8	10.1	13.9
**1g**	25.3	12.1	13.6
**1h**	20.2	10.7	17.9
**1i**	18.6	10.3	17.6

The steric effects of the alkene moiety were studied by the analysis of the effect on the TS barriers in methyl-substituted enynes **1e**–**1g**. The presence of a methyl group in the alkenyl moiety had a different effect depending on the diastereomer of the starting enyne. On one hand, both *Z*-methyl-substituted enyne **1e** and *E*-methyl-substituted enyne **1f** displayed similar activation barriers for the alkene insertion (**TS1**) but significantly higher barriers than **1a**. However, the barrier for the CO insertion was lower in the case of the *E*-stereoisomer (**1f-TS1**) compared to that of the unsubstituted enyne (**1a-TS1**) and *Z*-stereoisomer (**1e-TS1**). On the contrary, a lower barrier for reductive elimination was computed for **1e** and **1f**. The higher barrier for **TS1** can be attributed to the steric effects of the methyl group with a coordinated CO. In **1e-TS1**, the distances between the hydrogen atoms in the methyl group and oxygen atoms in the coordinated CO are 2.67 and 2.87 Å, respectively, which are shorter than those in **1f-TS1**, with distances of 3.07 and 2.52 Å, respectively. A closer inspection of both optimized TS structures for the CO insertion reveals that the methyl group and the coordinated CO are closer in enyne **1e**, but the transition state structure for **1f-TS2** shows a strong interaction of the methyl group with the inserted CO (distance H···C of 2.58 Å), which may hinder the CO insertion step for *Z*-methyl-substituted enyne **1e**. The energy difference for C–C bond formation in the reductive elimination of *Z*-methyl-substituted enyne **1e** was 1.5 kcal/mol higher than that of the *Z*-methyl-substituted enyne **1e**, probably due to the eclipsed conformation of the methyl group and newly inserted CO. The PKR of enynes **1e** and **1f** was assayed experimentally, yielding bicyclic products **2e** and **2f** in 15 and 40% yields, respectively.^17^ When evaluating the reactivity of dimethyl-substituted enyne **1g**, the activation barrier for the alkene insertion (**TS1**) was found to be significantly higher (25.3 kcal/mol), in agreement with previous reports, showing that trisubstituted alkenes are less reactive or, in some cases, unreactive substrates in the Pauson–Khand reaction.^36,37^ A higher barrier for CO insertion (**1g-TS2**) was also observed due to the crowded environment around the alkene by the two methyl groups.

Finally, substitutions on the alkyne moiety had a negative effect on reactions involving the alkyne counterpart, i.e., the alkene insertion (**TS1**) and the final reduction elimination (**TS3**). Thus, *N*-tethered enyne **1h** bearing a methyl-substituted alkyne had a higher activation barrier for alkene insertion and reductive elimination, whereas the CO insertion step was found to have similar activation energy to unsubstituted enyne **1a**. The situation was different for *N*-tethered enyne **1i** bearing a phenyl-substituted alkyne. In this case, a lower activation barrier was calculated for the alkene insertion step, which can be attributed to the polarization effect of the aromatic ring on the alkyne. On one hand, the barrier for CO insertion to alkenyl C7 was similar to that of **1a** and was higher than the barrier for the alkene insertion. On the other hand, the reductive elimination went through a TS slightly higher in energy when compared to unsubstituted enyne **1a**, as also observed for enyne **1d** bearing a strong electron-withdrawing group (−CF_3_).

## Conclusions

In summary, DFT calculations using the M11 functional with 6-311+G(d,p) including solvent effects by PCM have been used to study the mechanism of the Co_2_(CO)_8_-mediated Pauson–Khand reaction of asymmetric *N*-tethered enynes. The stereoselectivity-determining step was the intramolecular alkene insertion into the carbon–cobalt bond. Theoretical calculations indicate that *Re-*face insertion of the alkene is favored over the *Si*-face insertion, leading to the *R* configuration at C6, which is attributed to enhanced *N*-Bus---CF_3_ gauche interactions in the *Si*-face insertion as supported by NCI analysis and enhanced strain from activation/strain analysis. Moreover, the *Re-*face approach of the alkene promotes greater orbital mixing interaction between the alkyne and the Co_2_(CO)_5_ moiety. This step was also found to be the rate-determining step of the whole process. The presence of a methyl fluorinated group at the asymmetric carbon of the enyne had a positive effect by accelerating the alkene insertion. However, the introduction of fluorine or fluorinated groups on the alkene or alkyne moiety had a negative effect on the barriers of the TS for CO insertion and reductive elimination. Thus, from the results inferred from this computational study, fluorinated groups at the alkyne are not recommended, as they increase the reaction rate. These results provide a theoretical guide of great interest for the design of more reactive enynes as starting materials in Co_2_(CO)_8_-mediated Pauson–Khand reactions.

## Supporting Information Available

Full Gaussian 16 citation; computational methods; and cartesian coordinates for all reported structures (PDF)
