# Supplementary material for: Computational Study on the Co-Mediated Intramolecular Pauson–Khand Reaction of Fluorinated and Chiral N-Tethered 1,7-Enynes
Source: Organometallics. 2022 Sep 2;41(18):2525–34. doi: 10.1021/acs.organomet.2c00227 (PMC9516775; doi:10.1021/acs.organomet.2c00227)
Supplement: Supplementary file 1 — om2c00227_si_001.pdf [file om2c00227_si_001.pdf]

Supporting Information

A Computational Study on the Co-mediated Intramolecular Pauson-Khand Reaction of Fluorinated and Chiral *N*-tethered 1,7-Enynes

Jorge Escorihuela [1,*] and Lawrence M. Wolf [2,*]

1 Departamento de Química Orgánica, Facultad de Farmacia, Universitat de València, Av. Vicent Andrés Estellés s/n, 46100 Burjassot, València, Spain.

2 Department of Chemistry, University of Massachusetts–Lowell, 1 University Avenue, Lowell, Massachusetts 01854, United States of America

Email: jorge.escorihuela@uv.es (J.E.); Lawrence_Wolf@uml.edu (L.M.W.)

Table of Contents

1. Complete reference for Gaussian 16. S3

2. Computational details. S3

3. Optimized structures for PKR of compounds **1a** and **1a'**. S4

1. Complete reference for Gaussian 16.

Gaussian 16, Revision B.01, Frisch, M. J.; Trucks, G. W.; Schlegel, H. B.; Scuseria, G. E.; Robb, M. A.; Cheeseman, J. R.; Scalmani, G.; Barone, V.; Petersson, G. A.; Nakatsuji, H.; Li, X.; Caricato, M.; Marenich, A. V.; Bloino, J.; Janesko, B. G.; Gom-perts, R.; Mennucci, B.; Hratchian, H. P.; Ortiz, J. V.; Izmaylov, A. F.; Sonnenberg, J. L.; Williams-Young, D.; Ding, F.; Lippa-rini, F.; Egidi, F.; Goings, J.; Peng, B.; Petrone, A.; Henderson, T.; Ranasinghe, D.; Zakrzewski, V. G.; Gao, J.; Rega, N.; Zheng, G.; Liang, W.; Hada, M.; Ehara, M.; Toyota, K.; Fukuda, R.; Ha-segawa, J.; Ishida, M.; Nakajima, T.; Honda, Y.; Kitao, O.; Nakai, H.; Vreven, T.; Throssell, K.; Montgomery, J. A., Jr.; Peralta, J. E.; Ogliaro, F.; Bearpark, M. J.; Heyd, J. J.; Brothers, E. N.; Kudin, K. N.; Staroverov, V. N.; Keith, T. A.; Kobayashi, R.; Normand, J.; Raghavachari, K.; Rendell, A. P.; Burant, J. C.; Iyengar, S. S.; Tomasi, J.; Cossi, M.; Millam, J. M.; Klene, M.; Adamo, C.; Cammi, R.; Ochterski, J. W.; Martin, R. L.; Morokuma, K.; Farkas, O.; Foresman, J. B.; Fox, D. J. Gaussian, Inc., Wallingford CT, 2016.

2. Computational details.

All DFT geometry optimizations were performed with the M11 functional and using the SDD basis set for Co and the 6-311+G(d,p) basis set for the other atoms as implemented within the Gaussian 16 series of programs. Solvent effects were included with the conductor-like polarizable continuum model (CPCM) to mimic the solvent during both geometry optimizations and vibrational analysis. All energies presented for the reactant complex (RC), transition state (TS), and product (P) are given in Hartree. The vibrational frequencies were computed at the same level of theory as for the geometry optimizations to confirm whether each optimized structure is an energy minimum or a transition state, and to evaluate the electronic and thermal Free Energies and thermal corrections at 298 K. Vibrational frequency calculations were performed at the same level of theory used for optimization. All transition states were verified to have only one negative eigenvalue in the Hessian matrix, describing the motion along the reaction coordinate. In addition, intrinsic reaction coordinate (IRC) calculations were performed at the same level of theory to verify the expected connections of the first-order saddle points with the local minima found on the potential energy surface.

3. Optimized structures for PKR of compounds 1a and 1a'.

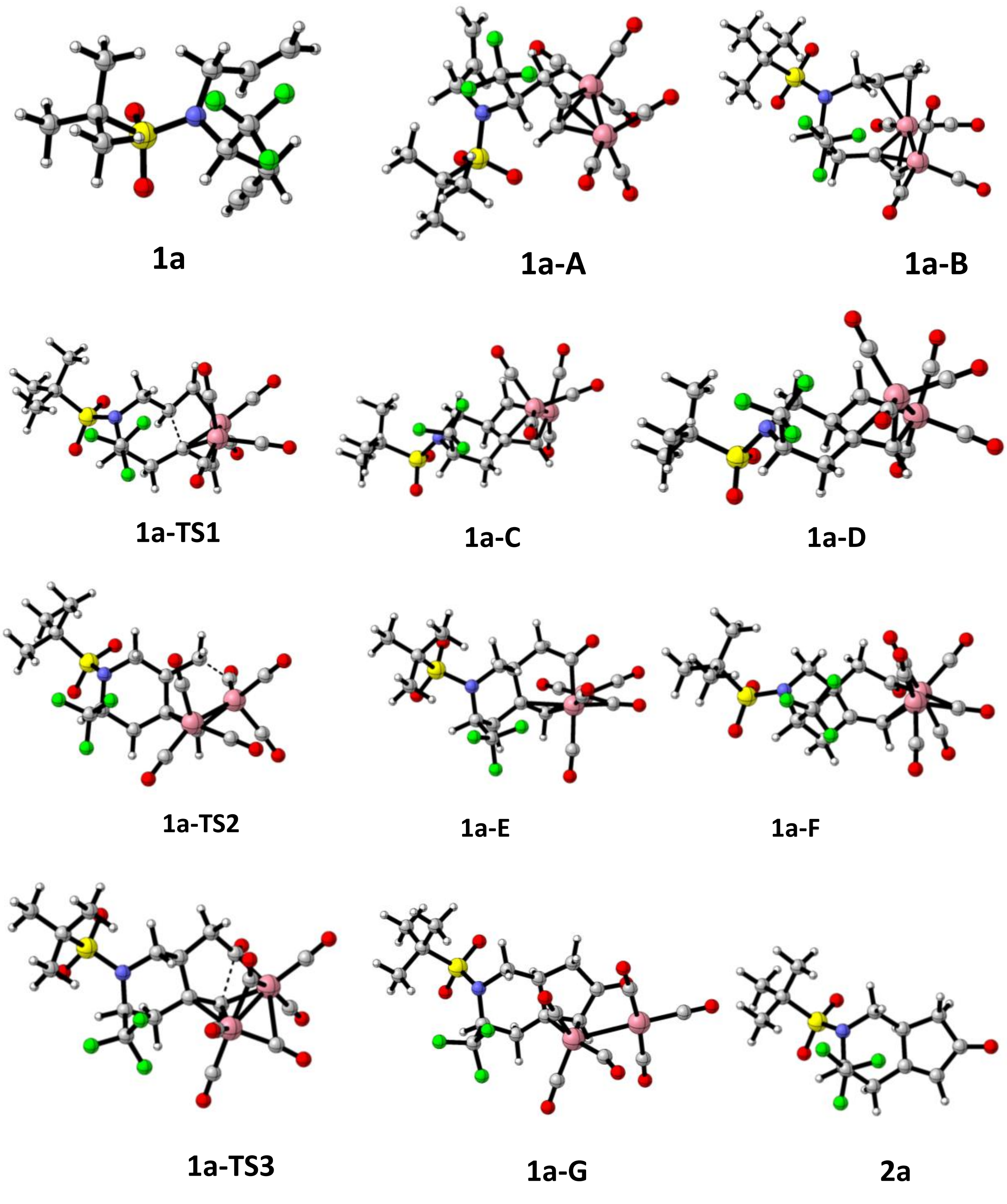

Figure S1. Optimized structures for PKR of **1a**.

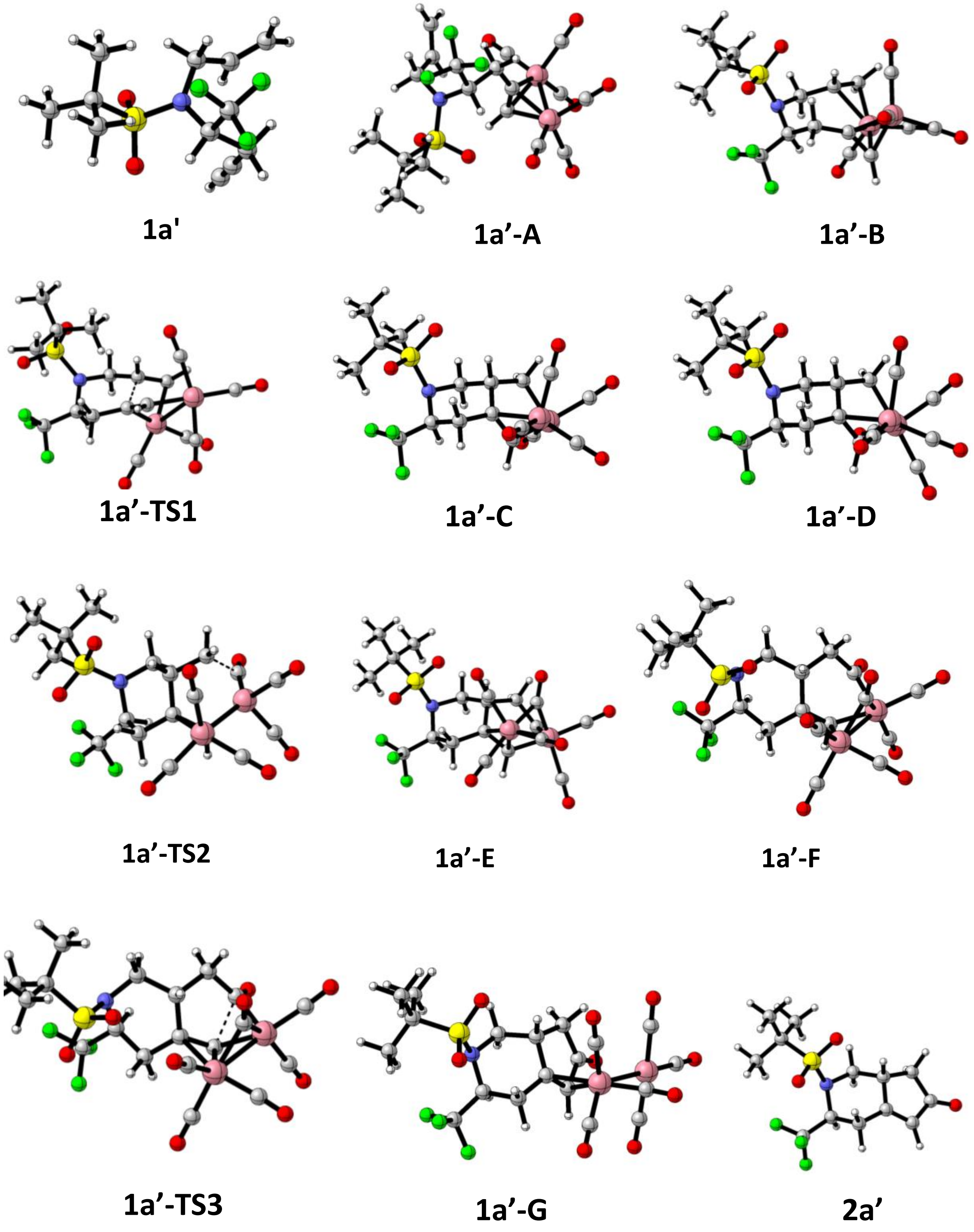

Figure S2. Optimized structures for PKR of **1a’**.

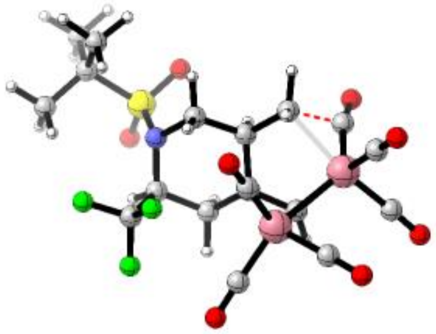

View 1 of TS for CO insertion (CO axial bottom)

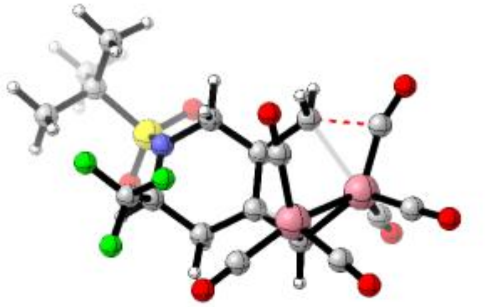

View 1 of TS for CO insertion (CO equatorial back)

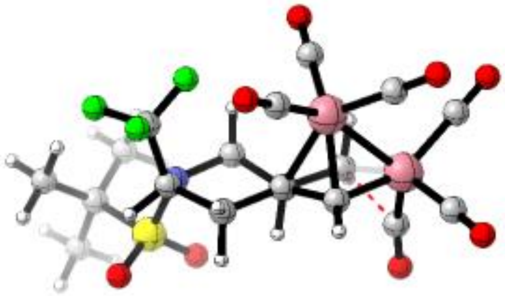

View 2 of TS for CO insertion (CO axial bottom)

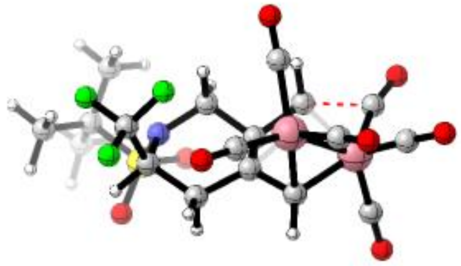

View 2of TS for CO insertion (CO equatorial back)

Figure S3. Optimized structures for different TS of the alkene insertion.
